# Molecular markers in *plasmodium falciparum *linked to resistance to anti-malarial drugs in samples imported from Africa over an eight-year period (2002-2010): impact of the introduction of artemisinin combination therapy

**DOI:** 10.1186/1475-2875-11-100

**Published:** 2012-03-30

**Authors:** Aranzazu Amor, Carlos Toro, Amalia Fernández-Martínez, Margarita Baquero, Agustín Benito, Pedro Berzosa

**Affiliations:** 1 Department of Microbiology and Parasitology, Hospital Carlos III, C/Sinesio Delgado 10, Madrid 28029, Spain; 2 Malaria Laboratory, National Centre of Tropical Medicine, Carlos III Institute of Health, C/Melchor Fernández Almagro 3, pabellón 13, Madrid 28029, Spain

## Abstract

**Background:**

Drug resistance is a major problem to control *Plasmodium falciparum *infection in endemic countries. During last decade, African countries have changed first-line treatment to artemisinin-based combinations therapy (ACT); sulphadoxine-pyrimethamine (SP) is recommended for Intermittent Preventive Therapy (IPT). Molecular markers related to *P falciparum *resistance were analysed for the period of transition from SP to ACT, in isolates imported from Africa.

**Methods:**

A first group of samples was taken in the period between June 2002 and June 2006 (n = 113); a second group in the period between November 2008 and August 2010 (n = 46). Several alleles were analysed by nested PCR-RFLP: 51, 59, 108, 164, in the *pfdhfr *gene; 436, 437, 540, 581, in the *pfdhps *gene; 86, 1246, in the *pfmdr1 *gene and 76, in the *pfcrt *gene. The prevalence of alleles in the groups was compared with the chi-squared or Fisher's exact tests.

**Results:**

The *pfdhfr *N51I, C59R and S108N were over to 90% in the two groups; all samples had the I164. In the *pfdhps*, 437 G and 581 G, increased up to 80% and 10.9% (*p *= 0.024), respectively in the second group. The 540 G decreases (24% to 16.%) and the 436A disappears at the end of the follow-up (*p *= 0.004) in the second group. The 76I-*pfcrt *stayed over 95% in the two groups. Prevalence of 86Y-*pfmdr1 *decreased over eight years.

**Conclusions:**

Pharmacological pressure affects the resistance strains prevalence. As for SP, the disappearance of 436A and the decrease in 540 G suggest that these mutations are not fixed. On the other hand, studies carried out after ACT introduction show there was a selection of strains carrying the SNPs N86Y, D1246Y in *pfmdr1*. In this work, the prevalence of *pfmdr1- *D1246Y is increasing, perhaps as a result of selective pressure by ACT. Continued surveillance is essential to monitor the effectiveness of treatments.

## Background

Malaria treatment is an essential tool to control *Plasmodium falciparum *disease in endemic countries. Resistance to currently available drugs is one of his main problems [1]. Several mutations linked to resistance have been described in different genes of *P. falciparum *genome. These mutations take place spontaneously in the parasite, though pharmacological pressure is one of the most important factors involved in their spread [2].

Resistance to chloroquine appears nowadays in all regions where *P. falciparum *is present in Africa [3]. The main determinant of chloroquine resistance is the K76I mutation of the *pfcrt *gene, which left to in vivo and in vitro resistance [4]. In addition, the N86Y and D1246Y mutations in the *pfmdr1 *gene, together with the occurrence of mutations in the *pfcrt *gene, reduce susceptibility to chloroquine [5].

Use of sulphadoxine-pyrimethamine (SP) began in Africa in the early 1980s and was adopted as first-line treatment for non-severe malaria in many sub-Saharan countries. In the last decade, the high resistance levels reached across the continent have led to a change in treatment policies. SP is currently recommended by the World Health Organization (WHO) for intermittent preventive therapy (IPT) in pregnant woman (IPTp) and infants (IPTi) [6].

Loss in efficacy of either component of SP brings about a reduction in the efficacy of the combination [7]. Resistance to pyrimethamine is related with single nucleotide polymorphisms (SNPs) at codons A16V, N51I, C59R, S108N/T and I164L of the *pfdhfr *gene. Although the C50R mutation is usually found in South America, it has recently been described in Africa [8]. The I164L mutation is hardly ever seen in Africa, and its association with resistance in this continent is doubtful [9]. In the *dhps *gene, five SNPs, namely, S436A/F, A437G, K540E, A581G and A613S/T, have been reported to be linked to *P. falciparum *resistance to sulphadoxine. An increase in the number of mutations in both, *pfdhfr *and *pfdhps*, genes leads to an increase in clinical resistance. In Africa, the *pfdhfr *triple mutant, 51I-59R-108 N, together with the *pfdhps *double mutant, 437 G-540E, the so called *dhfr/dhps *quintuple mutation predicts treatment failure with SP [10].

Nowadays, artemisinin combination therapy (ACT) is used as a first-line treatment in uncomplicated malaria in African countries with artemether-lumefantrine (AL) and artesunate-amodiaquine (AS/AQ) being the combinations used [11]. The SNPs in the *pfcrt *and *pfmdr1 *genes are related with the efficacy of amodiaquine, which is structurally related to chloroquine although in vivo and in vitro data suggest that cross-resistance between both molecules is incomplete [12]. The wild-type alleles of the *pfmdr1 *gene, N86 and D1246, are linked to a decrease in the in vitro response to lumefantrine and artemisinin [13]. After the introduction of AL, an in vivo association has been established with re-infections by strains with wild-type *pfmdr1 *alleles and the wild-type *pfcrt *allele, K76 [14].

The Hospital Carlos III (Madrid, Spain) is a tropical disease reference centre. Its Tropical Medicine and Paediatrics Departments, attends to immigrants and travellers. SNPs in *P. falciparum *linked to resistance were analysed in patients proceeding from African countries. Samples were collected over a period of eight years, during a transition phase from high SP coverage to treatment with ACT. This study sought to ascertain the prevalence of anti-malarial drug resistant strains during this period, and measure the impact of pharmacological pressure.

## Methods

### Biological samples

**Figure 1:**
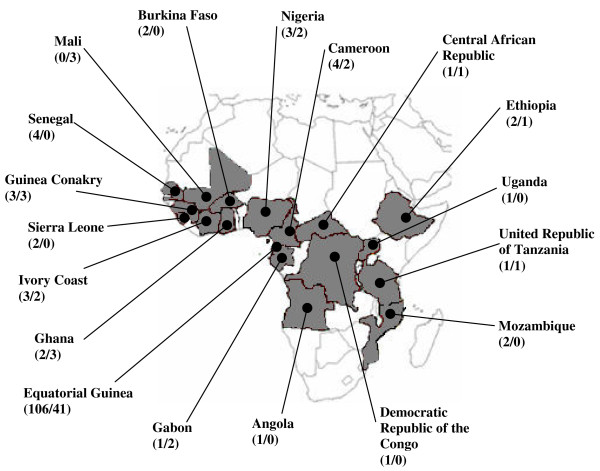
**African countries of origin of samples**.

Blood samples were collected from 200 patients with *P falciparum *infection, who had come to Spain from eighteen African countries (Figure 1). After microscopic diagnosis, identification to species level was performed by polymerase chain reaction (PCR). DNA was extracted from 200 μl of blood using QIAamp DNA Blood Minikits^® ^(QIAGEN, Hilden, Germany). Samples were classified in two groups, the first including those collected between June 2002 to June 2006, and the second one including those collected between November 2008 to August 2010.

### Molecular assays

Multiplex PCR was performed to diagnose the malaria species [15]. Nested polymerase chain reaction-restriction fragment length polymorphism (PCR-RFLP) was used to analyse the presence/absence of mutations at codons 51, 59, 108 and 164 of the *pfdhfr *gene, codons 436, 437, 540 and 581 of the *pfdhps *gene, codons 86 and 1246 of the *pfmdr1 *gene, and codon 76 of the *pfcrt *gene [16]. In the same way of other works,, mixed populations were deemed mutants [17].

### Statistical analysis

All analyses were performed using the SPSS statistical software package, version 15.0. The prevalence of mutations in the groups was compared using the chi-squared (*χ*2) or Fisher's exact test. Statistical significance was set at *p *≤ 0.05.

## Results

The typing efficiency for each codon was as follows: 100% of samples for the *pfdhfr-*51- 59; 99.5% for *pfdhfr-*108 and *pfdhps*-436,-437; 99% for *pfdhps-*540-581; 98% for *pfcrt-*76; 97.5% for *pfmdr1-*1246*; *96% for *pfmdr1-*86 and 95% for *pfdhfr-*164.

Changes in the prevalence of typed alleles over the two periods are shown in the Table 1.

**Table 1:** Molecular markers of resistance (%)

n = 200									
	**I** **2002-2005** **(n = 139)**		**II** **2008-2010** **(n = 61)**			**I**	**II**	**I**	**II**

	**W**	**M**	**W**	**M**	**p**	Combined genotypes			

***pfdhfr-*51**	**4.3**	**95.7**	**8.2**	**91.8**	0.3	***pfdhfr*-51I-59R-108N**		Cuadruple ***pfdhf-pfdps***	
			
***pfdhfr-*59**	**5**	**95**	**8.2**	**91.8**	0.5			**51I-59R-108N-437G**	

***pfdhfr-*108**	**1.4**	**98.6**	**1.6**	**98.4**	1	**90.8**	**81.3**	**53.4**	**63.3**
			
***pfdhfr-*164**	**100**	**0**	**100**	**0**	1			Quintuple ***pfdhf-pfdps***	

***pfdhps-*436**	**87.7**	**12.3**	**100**	**0**	**0.004**	***pfdhps*-437G**		**51I-59R-108N-437G-540E**	
			
***pfdhps-*437**	**29**	**71**	**16.4**	**83.6**	**0.05**			**18.4**	**16.3**

***pfdhps-*540**	**75.4**	**24.6**	**83.3**	**16.7**	0.2	**46.7**	**60**	**51I-59R-108N-437G-581G**	
			
***pfdhps-*581**	**97.8**	**2.2**	**90**	**10**	**0.02**			**1**	**8.2**

***pfmdr1-*86**	**48.5**	**51.5**	**58.6**	**41.4**	0.1				
				
***pfmdr1-*1246**	**64.9**	**35.1**	**91.8**	**8.2**	**0.0001**				
				
***pfcrt-*76**	**4.4**	**95.6**	**3.4**	**96.6**	1				

Wild-type (**W) **and mutant-type (**M**) alleles; **I**: 2002-2006; **II**: 2008-2010

### Pfdhfr-pfdhps

The frequency of the combination of *pfdhfr *mutated alleles *with *more than one mutation increased across the Continent, with the most frequent being the *pfdhfr *triple mutant, 51I-59R-108 N. In the *pfdhps *gene, the most frequent genotype was 437 G, followed by the double mutant, 437 G-540E, though this one decreased slightly owing to the fall in the 540E mutation. The *pfdhps *double mutant, 437 G-581 G, also increased (*p *= 0.03).

Mutations in both *pfdhfr *and *pfdhps *genes were observed in 76% of patients (n = 152). At the end of the study, the most prevalent were: the quadruple mutant, 51I-59R-108 N-437 G, followed by the quintuple mutant, 51I-59R-108 N-437 G-540E, and the quintuple ones 51I-59R-108 N-437 G-581 G, which increases his prevalence (*p *= 0.03).

### Pfcrt

Mutation 76I of *pfcrt *keeps above 95% during the study period (Table 1).

### pfmdr1

The prevalence of wild-type alleles of the *pfmdr1 *gene, N86 and D1246, increased across the eight years.

## Discussion

In this study, although samples from 18 countries, patients from Central and West Africa accounted for 79.9% and 16.1% respectively of all, and those from the East of the continent were scarce (4%). Even so, there are few studies about changes molecular markers of resistance after large-scale deployment of ACT in the entire African Continent, in a long period of time. Malaria treatment is one of the mainstays of disease control programmes, and parasite resistance to anti-malarial drugs is one of its major problems. Current treatment policies are based on ACT, so as to reduce the loss of efficacy and emergence and spread of resistance resulting from pharmacological pressure and migratory movements, among other factors [18]. Surveillance during the period of treatment policies change is essential, to evaluate its impact: in cases where resistance has not yet become permanent in a population, prevalence of resistance-related alleles should decline if pharmacological pressure ceases [19]. However, up to now, molecular markers related to SP resistance appear to show no reduction after the introduction of ACT [6]. Indeed, an increase has even been observed in some regions; this has been attributed to the widespread use of other drugs, such as cotrimoxazole [20], which have cross-resistance with SP, and to the fact that the combination is widely used outside the official sector [21]. In this way, in this study the *pfdhfr *triple mutant, 51I-59R-108 N, became established. With regard to mutations linked to sulphadoxine resistance, in spite of a significant disappearance of 436A, there are a significant increase of the 437 G allele and, it should be stressed the emergence of mutant 581 G in Cameroon, two cases, Gabon, one case, and Equatorial Guinea, five cases, countries in which this mutation has never been previously detected. Also, a case of 581 G allele was also detected in Mali, where this mutation has been already described [22]. It has been noted that in areas where the *pfdhfr *triple and *pfdhps *double mutants are established, the emergence of mutation 581 G can endanger intermittent preventive therapies with SP. It has also been shown a selection of this mutated allele in woman receiving IPT [23,24]. At all events, to check whether significant changes are taking place in the prevalence of mutations linked to a decrease in the use of SP, and to monitor the appearance of mutations that, until now, have rarely been described in these regions (581 G), continued surveillance is needed to evaluate their impact on IPT [25,26].

For molecular markers related to anti-malarial drugs currently being used in Africa (*pfcrt *and *pfmdr1 *genes), a reduction in the prevalence of *pfcrt *76I and *pfmdr1 *N86Y mutations in response to a decrease in chloroquine use has been observed in some regions [27]. On the other hand, the wild-type *pfmdr *N86 and the *pfcrt *K76 genotypes are associated with lower susceptibility to dihydroartemisinin in vitro [12]. In addition, studies undertaken after the introduction of ACT have shown that there is a selection of strains carrying wild-type alleles, *pfmdr1 *N86, *pfmdr1*D1246 [28] and *pfcrt *K76 [15]. The first has been linked to a significant decrease in in vitro sensitivity to lumefantrine and an increase of reinfections in vivo after AL treatment, a finding that renders the use of lumefantrine advisable in areas where chloroquine-resistant mutations remain high [6]. In this way, the prevalence of *pfcrt *76I had risen to over 95% by the end of the study: nevertheless, the prevalence of wild-type *pfmdr1 *N86 shows a statistical tendency to increase and the wild-type *pfmdr1*D1246 raises until 91.8% ant the end of the study. It is possible that these results reflect the first step in ACT resistance.

## Conclusions

This study suggests the appearance of strains related with resistance to ACT. On the other hand, the study shows the existence of mutations in some regions in the African Continent who will put in danger the use of IPTp and IPTc. In vivo surveys are the gold standard for analysis of malaria therapy resistance, but it can be concluded that surveys like the one described in the current paper could be an essential tool to assess and follow up the long-term use and efficiency of ACT and IPT.

## Competing interests

The authors declare that they have no competing interests.

## Authors' contributions

AA: carried out the laboratory work, data collection and analysis of the results and wrote the manuscript. AMF: was involved in laboratory work. CT revised the manuscript. MB and AB contributed to reagents and helped the study. PB: was involved in laboratory work and revised the manuscript. All authors read and approved the final manuscript.
